# Recent advances and future avenues in understanding the role of adipose tissue cross talk in mediating skeletal muscle mass and function with ageing

**DOI:** 10.1007/s11357-021-00322-4

**Published:** 2021-02-02

**Authors:** Andrew Wilhelmsen, Kostas Tsintzas, Simon W. Jones

**Affiliations:** 1 MRC Versus Arthritis Centre for Musculoskeletal Ageing Research, School of Life Sciences, University of Nottingham, Queen’s Medical Centre, Nottingham, UK; 2 Institute of Inflammation and Ageing, MRC Versus Arthritis Centre for Musculoskeletal Ageing Research, Queen Elizabeth Hospital, The University of Birmingham, Birmingham, UK

**Keywords:** Skeletal muscle, Adipose tissue, Obesity, Cross talk, Ageing, Cytokines, Long non-coding RNAs, MicroRNAs, Cellular senescence

## Abstract

Sarcopenia, broadly defined as the age-related decline in skeletal muscle mass, quality, and function, is associated with chronic low-grade inflammation and an increased likelihood of adverse health outcomes. The regulation of skeletal muscle mass with ageing is complex and necessitates a delicate balance between muscle protein synthesis and degradation. The secretion and transfer of cytokines, long non-coding RNAs (lncRNAs) and microRNAs (miRNAs), both discretely and within extracellular vesicles, have emerged as important communication channels between tissues. Some of these factors have been implicated in regulating skeletal muscle mass, function, and pathologies and may be perturbed by excessive adiposity. Indeed, adipose tissue participates in a broad spectrum of inter-organ communication and obesity promotes the accumulation of macrophages, cellular senescence, and the production and secretion of pro-inflammatory factors. Pertinently, age-related sarcopenia has been reported to be more prevalent in obesity; however, such effects are confounded by comorbidities and physical activity level. In this review, we provide evidence that adiposity may exacerbate age-related sarcopenia and outline some emerging concepts of adipose-skeletal muscle communication including the secretion and processing of novel myokines and adipokines and the role of extracellular vesicles in mediating inter-tissue cross talk via lncRNAs and miRNAs in the context of sarcopenia, ageing, and obesity. Further research using advances in proteomics, transcriptomics, and techniques to investigate extracellular vesicles, with an emphasis on translational, longitudinal human studies, is required to better understand the physiological significance of these factors, the impact of obesity upon them, and their potential as therapeutic targets in combating muscle wasting.

## Introduction

Beyond middle-age, skeletal muscle mass, and strength decline by 1–2% and 1–5% per year, respectively [1–3]. Skeletal muscle tissue is the human body’s principle protein bank, and given the absence of a protein pool for storage, it is critical for maintaining protein status via the delicate regulation of its turnover [4, 5]. Indeed, the maintenance of skeletal muscle mass, quality, and function with ageing is multifactorial and necessitates a balance of regulatory processes in response to inherent biological ageing, physical in/activity, injury, or illness [6]. These processes include the generation of muscle via adult myogenesis (comprising the activation of muscle satellite cells, the proliferation of myoblasts; the withdrawal of myoblasts from the cell cycle, their subsequent differentiation and fusion into multinucleated myofibres) [7, 8], the repair and remodelling of muscle tissue [9] and its inter- and intracellular content [10], the interaction between skeletal muscle and the nervous system via motor neurones [11], and the interplay between synthesis and breakdown of muscle protein [12, 13].

Sarcopenia, broadly defined as the age-related decline in skeletal muscle mass, quality, and function, is the product of a negative balance of these muscle regulatory processes and is associated with an increased likelihood of adverse health outcomes [14]. Sarcopenia has been reported to be more prevalent in adults with obesity [15]. Adiposity exacerbates sarcopenia, increases fat infiltration in muscle, and reduces physical function [14, 16, 17]. The state of concurrent obesity and sarcopenia is considered by some to constitute a discrete condition, termed sarcopenic obesity; however, there is a lack of consensus surroundings its definition and diagnostic criteria [18–20]. Individuals presenting with sarcopenic obesity may have greater absolute muscle mass than is typical of individuals with sarcopenia, but the quality and function of that muscle (including muscle strength) are reduced, which may exacerbate muscle deterioration with ageing [21–24].

Critically, as our population becomes increasingly aged and obese, the ramifications of obesity on the mass and function of skeletal muscle with age warrant rigorous investigation [25, 26]. In this review, we outline some emerging conduits of adipose-skeletal muscle communication in the context of sarcopenia and obesity including the secretion and processing of novel myokines and adipokines, as well as the significance of cellular senescence on the adipose tissue secretome, and the role of extracellular vesicles and non-coding RNAs in mediating inter-tissue cross talk. We principally focus on sarcopenia and obesity as two distinct but often converging conditions. We also present evidence investigating the phenomenon of sarcopenic obesity and discuss the myriad ways that obesity may interact with sarcopenia to the detriment of muscle mass, quality, and function.

## Adipose tissue as an endocrine organ

Once considered a passive energy reservoir, the discovery of the adipose-secreted factors adipsin and leptin confirmed adipose tissue as an endocrine organ [27–29]. Following the initial observation that obese adipose tissue has increased expression and secretion of tumour necrosis factor-alpha (TNF-α), it has been shown that obesity leads to the accumulation of macrophages within adipose tissue, which promote the production and secretion of several pro-inflammatory cytokines [30, 31]. Moreover, cellular senescence in adipose tissue, common to both ageing and obesity, is now understood to contribute to inflammation, aberrant cytokine production, and metabolic dysfunction [32]. Adipose tissue is now known to participate in a broad spectrum of inter-organ communication [33] and excessive adiposity conveys pleiotropic effects on its endocrine and metabolic function, contributing to pathophysiological consequences with ageing [34, 35]. Importantly, this has to be considered in the context of evidence from studies that demonstrated a capacity for healthy adipose tissue to exert favourable effects on skeletal muscle that further supports the physiological importance of cross talk between the two tissues [36].

## Inter-tissue communication via multiple means

Historically, communication between cells was thought to be mediated by cell-cell contact or by the extracellular secretion of molecules, principally cytokines, a broad family of small secreted proteins, which can confer autocrine, paracrine, and endocrine effects on multiple tissues [37]. While cytokines can be secreted discretely, accumulating evidence has demonstrated that they may also be transported by the secretion and transfer of extracellular vesicles (EVs) and that certain cytokines may also serve to regulate the packaging and trafficking of these EVs [38]. EVs, principally divided into exosomes and microvesicles, are respectively released into the extracellular environment from endosomal and plasma membrane origins and are reviewed elsewhere [39, 40]. Alongside cytokines, EVs contain a broad array of cargo including proteins, lipids, organelle components, and myriad non-coding RNAs, which have emerged as an important communication channel between tissues [41]. Pertinently, the discrete and EV-mediated secretion and processing of novel cytokines, long non-coding RNAs (lncRNAs), and microRNAs (miRNAs), from adipose and skeletal muscle tissue, have been implicated in the regulation of skeletal muscle mass, function, and pathologies, in the context of obesity and ageing [42, 43] (Table 1).

**Table 1:** Summary of adipose- and skeletal muscle-derived communication (cross talk) factors involved in mediating muscle mass and function in ageing

Communication factor	Predominant expressive Tissue	Significance and function in skeletal muscle mass Regulation	Proposed mechanisms	Effect of ageing	Key references
*Cytokines*					
Resistin	Adipose	Impairs myoblast differentiation in vitro*.*	Activation of classical NF-κβ pathway.	More pronounced effect on myotubes from older adults. Plasma Resistin concentration inversely correlated with muscle torque in older adults.	[18, 44–46,47]
IL-15	Skeletal Muscle	Promotes myoblast differentiation and protects against TNF-α-induced damage in vitro*.*	Decelerates proteolysis through suppression of E3 ubiquitin ligases MAFbx and MuRF-1.	Plasma concentration and muscle mRNA expression elevated in older adults. Lower plasma concentration in sarcopenic than non-sarcopenic older adults.	[48–50]
Adiponectin	Adipose	Activates satellite cells and promotes their motility in vitro. Drives myoblasts to exit cell cycle and promotes differentiation. Protects against muscle atrophy in mice.	Activation of RAC1 and expression of Snail and Twist transcription factors. Induces MHC expression and activates p38, Akt and AMPK pathways. Suppresses MAFbx and MuRF-1.	Lower plasma adiponectin in sarcopenic than non-sarcopenic older adults. Not different between healthy, physical activity-matched older and younger adults.	[51–55]
Leptin	Adipose	Promotes myoblast proliferation and prevents premature terminal differentiation in vitro. Increases muscle mass and fibre size in aged mice and prevents muscle atrophy in leptin-deficient mice.	Activates JAK2, promoting phosphorylation of IRS1 and IRS2, PI3K activity, and phosphorylation of Akt and p38 MAPK.	Circulating leptin increases with ageing and obesity, and is greatest in the sarcopenic-obese state, but may be downregulated in the severely-frail elderly. Upregulation of leptin is associated with resistance to its action in peripheral tissues and may thus inhibit its muscle-promoting effects.	[56–63]
Lipocalin-2	Adipose	Increased expression in regenerating mouse muscle and Pax-7^+^ satellite cells. Global knockout impairs satellite cell activation and muscle regeneration.	Involvement in MMP system, with Lcn2 knockout promoting fibrosis and impairing MMP-9 activity during muscle regeneration.	Currently unclear. Lcn-2 expression is induced by inflammatory stimuli, and correlates with inflammatory markers, so plausibly elevated with inflammaging.	[64–67]
Myostatin	Skeletal Muscle	Potent negative regulator of skeletal muscle mass. Impairs myogenic processes in vitro*;* suppressing satellite cell activation; impairing myoblast proliferation and differentiation, while knockout increases muscle mass in vivo*.*	Canonical TGF-β signalling activates SMAD2/3 transcription factors, inhibiting hypertrophic and promoting atrophic signalling pathways.	Increased myostatin protein and mRNA expression in aged muscle. Unclear whether upregulation is caused by ageing per se or age-related physical inactivity.	[68–76]
*lncRNAs*					
H19	Skeletal Muscle	Upregulated during myoblast differentiation. H19 knockdown in vitro and knockout in mice decreases myoblast differentiation.	Trans-regulatory function in muscle differentiation and regeneration, mediated by miR-675-3p and miR-675-5p via suppression of BMP/TGF-β pathway.	Currently unclear. Differentially expressed in myositis patients, relative to healthy controls. Plausible involvement in age-related muscle wasting and inflammaging.	[77, 78]
MALAT1	Skeletal Muscle	Upregulated during myoblast differentiation in vitro. In vitro knockdown accelerated myoblast differentiation and knockout in mice enhanced muscle regeneration. Conversely, silencing MALAT1 inhibited myoblast differentiation in vitro*.*	Recruits Suv39h1 to MyoD-binding loci, suppressing target gene expression. Competitively binds miR-133, de-repressing SRF, promoting muscle-specific gene expression in myoblasts.	Currently unclear. Myostatin greatly suppresses MALAT1 expression. Plausible that elevated myostatin abundance with ageing may suppress MALAT1 expression which may influence myogenesis.	[79, 80]
PVT1	Skeletal Muscle	Involved in modulating apoptosis and muscle atrophy. In vivo suppression of PVT1 attenuates myofibre atrophy under muscle wasting conditions.	PVT1 downexpression destabilises c-Myc, up-regulating the anti-apoptotic protein BCL-2, which is centrally involved in regulating apoptosis and atrophy.	Currently unclear. Implicated in a variety of inflammatory disease states including osteoarthritis and obesity. Plausibly similarly implicated in inflamed ageing muscle.	[81–83]
lncMyoD	Skeletal Muscle	Activated during myoblast differentiation. Enacts anti-proliferative effects to promote a permissive environment for myoblast differentiation.	Binds to IMP2, blocking IMP-mediated shuttling of proliferation-promoting RNAs; inhibiting their translation.	Currently unclear. Upregulated during disuse atrophy but not systemic muscle wasting. Plausible significance in age-related physical inactivity and immobilisation.	[84, 85]
*miRNAs*					
miR-1	Skeletal Muscle	Promotes myoblast differentiation. Combined administration with miR-133 and miR-206 accelerates muscle regeneration in vivo and promotes myoblast differentiation in vitro.	Post-transcriptionally downregulates the muscle gene transcriptional repressor HDAC4.	Currently unclear. However elevated skeletal muscle miR-1 expression in murine models of progeroid ageing.	[86–88]
miR-33a	Currently unclear	Impairs myoblast proliferation in vitro*.*	Targets IGF-1, Follistatin and Cyclin-D1 to inhibit proliferation by suppressing the PI3K/Akt/mTOR pathway.	Lower plasma miR-33a in older adults.	[279, 283–285]
miR-133a/b	Skeletal Muscle	Inhibits myoblast differentiation and promotes proliferation.	Represses the expression of SRF.	Downregulation in muscle of miR133a and miR-133b in healthy older men. Conversely, pri-miRNA-133a-1 and -a-2, but not their mature counterparts, are upregulated in elderly men.	[86, 87, 89, 90]
miR-206	Skeletal Muscle	Promotes myoblast differentiation.	MyoD activates miR-206 which targets and represses Follistatin-like-1 and Utrophin. Intermediary downstream effects unclear.	No difference in basal muscle expression of miR-206 between young and old adults, however greater exercise-induced increase in pri-miR-206 expression in older adults. Elevated muscle miR-206 in aged mice.	[56, 87, 90, 91]

## Senescence-associated secretory phenotype

Cellular senescence is an evolutionarily conserved ageing mechanism characterised by upregulation of cyclin-dependent kinase inhibitor genes, which in turn activate retinoblastoma protein to block cell cycle progression [92–94]. Senescence arises in response to cellular stress and damage, including metabolic insults [95, 96]. While this serves to restrain harmful growth and replication of damaged cells, senescent cells also secrete an array of cytokines, chemokines, growth factors, and matrix-remodelling proteases, collectively termed the senescence-associated secretory phenotype (SASP) [97, 98]. Senescent cells are resistant to apoptosis and are ordinarily cleared by the immune system but accumulate with ageing [99, 100]. Conditioned media from primary human pre-adipocytes with induced senescence via serial passage or irradiation demonstrate a SASP that is rich in pro-inflammatory interleukins, interferon gamma (IFN-γ), and TNF-α, which could negatively impact upon muscle regulation [101].

Accumulation of senescent cells within metabolic tissues, such as adipose and skeletal muscle, is deleterious to metabolic homeostasis [95, 102–104], and obesity may promote the senescent state [105]. Indeed, the adipose tissue of individuals with obesity displays elevated oxidative stress and accelerated telomere shortening [106, 107], which are purported to induce and promote senescence [108, 109]. It has been proposed that obesity and ageing drive excessive turnover of adipose progenitors which may contribute to the onset of their senescence and subsequent SASP, conveying both local and systemic adipose tissue dysfunction and inflammation [110].

The potential benefit of modulating cellular senescence has been recently investigated. Administration of Dasatinib and Quercetin, which target and eliminate senescent cells, to omental adipose tissue explants from middle-aged adults with obesity reduced the abundance of senescent cells and decreased pro-inflammatory cytokine secretion [111]. No study to date has directly investigated whether adipose tissue senescence is implicated in sarcopenia and whether this is amplified by obesity. However, the accumulation of intramuscular adipose tissue that is common to both ageing and obesity [112, 113], the pro-inflammatory adipose SASP observed in those states, and the ability of senescent cells to confer senescence to their microenvironment suggest that this relationship is plausible [17, 114]. To this effect, long-term studies into the implications of adipose senescence and the viability of anti-senescent therapeutics in sarcopenia are needed. It must be considered, however, that the human lifespan is vastly different to that of rodents, from whom most existing data is derived. Thus, any deleterious effects of cytotoxic therapies to remove senescent cells may be amplified over the course of human ageing [92].

## Cytokines as mediators of adipose-muscle cross talk

**Fig. 1:**
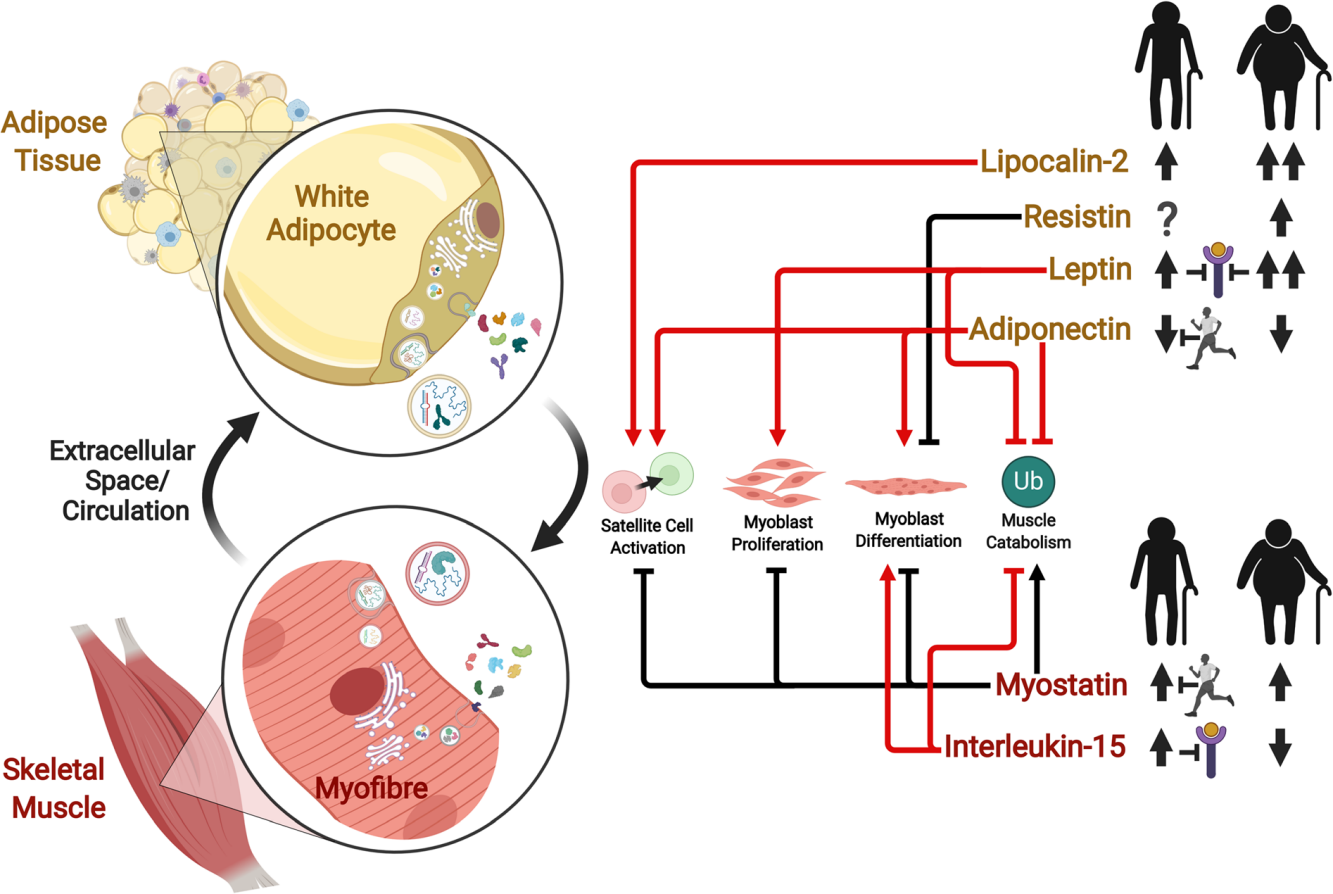
Cytokines secreted from skeletal muscle and adipose tissues with emerging significance in muscle-adipose cross talk and the regulation of skeletal muscle mass. The expression, secretion, extracellular-vesicle-mediated transport, and function of these cytokines may be perturbed by ageing and obesity, impairing normal muscle regulatory pathways. Processes regulating skeletal muscle mass have been condensed into four fundamental levels: activation of the muscle satellite cell pool; proliferation of myoblasts; differentiation of myoblasts into myotubes; and the ubiquitin (Ub) catabolic processes involved in the breakdown of muscle protein. Regardless of colour, arrows and block (inhibitory) lines indicate stimulatory and inhibitory effects, respectively, of a particular cytokine on these regulatory levels. Red lines (whether arrows or block lines) indicate pro-myogenic effects, which may act to preserve muscle mass, while black lines (whether arrows or block lines) indicate anti-myogenic effects which may confer adverse effects on muscle mass. The effects of ageing without obesity (silhouetted figure on the left) and ageing combined with obesity (silhouetted figure on the right) to increase or decrease the secretion and/or circulating abundance of these cytokines is indicated by thick black upward or downward-pointing arrows, respectively. Two upward arrows indicate a greater effect of obesity than the lean state on the relevant cytokine with ageing. A question mark indicates an unknown effect. An inhibitory line extending from a running person indicates that the proposed effect of ageing is offset when physical activity level is maintained with ageing. An inhibitory line extending to a cytokine receptor indicates that an increased abundance of that cytokine is associated with a reduction in expression of its receptor in skeletal muscle. Created with BioRender.com

The decline of skeletal muscle mass in various inflammatory states is associated with elevated production of classical pro-inflammatory cytokines, including TNF-α, interleukin (IL)-1 beta (IL-1β), IL-6, and IFN-γ [115]. The low-grade inflammation associated with ageing, termed “inflammaging,” is influenced not only by overproduction of pro-inflammatory mediators but also by perturbation of anti-inflammatory factors, such as IL-10 [116–119]. While far from being fully understood, the roles of such classical cytokines in ageing muscle have been reviewed elsewhere [115, 120–122]. Accordingly, here we focus on cytokines which are implicated in adipose-muscle cross talk with emerging significance in the regulation of skeletal muscle mass, namely resistin, IL-15, adiponectin, leptin, lipocalin-2, and myostatin (Fig. 1; Table 1).

### Resistin

Resistin was first identified in rodents as an adipocyte-secreted factor that is upregulated in obesity and impairs glucose tolerance, insulin action, and fatty acid handling in skeletal muscle [123–126]. While the significance and even existence of resistin in human adipocytes has been contentious [127], by analysing conditioned medium from subcutaneous adipose tissue (SAT) from lean and obese humans, rather than isolated adipocytes, we recently found it to be secreted from SAT and increased with obesity [47]. This highlights the importance of stromal and infiltrating immune cells to the inflammatory in vivo phenotype of adipose tissue [128].

Notably, incubation of primary human myotubes from lean individuals with SAT-conditioned media obtained from obese subjects produced thinner, less multinucleated myotubes than those exposed to lean SAT secretome, which is concordant with findings using co-culture systems of human myotubes and adipocytes isolated from visceral adipose tissue (VAT) [47, 129]. Pertinently, the deleterious effects of the obese SAT secretome were more pronounced in myotubes derived from older than younger individuals, suggesting ageing impairs the ability of muscle to withstand inflammatory challenges. Since resistin was prolifically secreted from obese SAT, further investigation revealed that a physiologically relevant concentration of resistin (5 ng mL^−1^) impaired myoblast differentiation through activation of the classical nuclear factor-kappa beta (NF-κβ) pathway, similarly producing thinner myotubes with reduced nuclear fusion [47]. This adverse effect of resistin was confirmed in experiments showing that its depletion from obese SAT secretome restored myogenesis [47]. Consistent with these findings, overexpression of resistin impairs C2C12 myoblast differentiation, resulting in thinner myotubes with reduced expression of desmin and myoglobin [44].

In vivo research on the effects of resistin on regulating skeletal muscle mass and function is scant. However, human plasma resistin concentrations correlate with inflammatory markers [130], have been reported to correlate with age [45], and in the elderly are inversely associated with quadriceps torque [46] and computed tomography determinants of abdominal muscle density (a marker of muscle quality and composition) [131]. Collectively, the limited literature suggests an emerging role for resistin in suppressing myogenic differentiation, particularly in older skeletal muscle, which appears to be driven by inflammation and obesity.

### Interleukin-15

IL-15, a four-helix bundle family cytokine, is produced by a variety of tissues, including skeletal muscle and adipose. Its mRNA expression is upregulated during the differentiation of C2C12 myoblasts and it is also expressed in pre- and post-differentiated 3T3-L1 adipocytes, albeit at a comparatively much lower level [132]. IL-15 mRNA expression in mouse epididymal adipose tissue and adipose tissue macrophages is increased in response to a high-fat diet (HFD) [133]. IL-15 was originally observed to have pro-myogenic effects on murine C2 skeletal muscle cells by promoting the accumulation of contractile proteins [134, 135]. Such effects may be mediated by supressing muscle proteolytic pathways. Indeed, ex vivo incubation of rat muscle tissue with recombinant IL-15 decreased the rate of proteolysis, without a change in total protein synthesis or tissue amino acid uptake [136]. Similarly, in vivo studies showed that septic mice pre-treated with IL-15 had lower expression of the proteolytic E3 ubiquitin ligases muscle atrophy F-box gene/atrogin-1 (MAFbx) and muscle ring finger-1 (MuRF-1), and IL-15 treatment of cachexic rats decelerated protein degradation and suppressed expression of ubiquitin proteolysis pathway components [48, 137].

We also reported that exposing primary myogenic cultures from young and elderly adults to recombinant IL-15 during differentiation resulted in thicker myotubes that were protected against differentiation impairment by TNF-α [49]. However, subcutaneous administration of recombinant IL-15 in rats for 7 days did not impact upon net muscle mass or protein content, although it increased turnover by accelerating both protein synthesis and breakdown rates and decreased white adipose tissue (WAT) mass by 33% [138]. Taken together, the literature supports a muscle-sparing effect of IL-15, particularly in conditions of dysregulated protein turnover such as cancer cachexia and possibly sarcopenia, through the suppression of muscle protein catabolism. However, IL-15 appears to have limited effects on muscle anabolism, particularly in healthy states.

We recently demonstrated in healthy older adults that IL-15 muscle mRNA expression and plasma concentration are elevated 2-fold and 1.5-fold, respectively, relative to younger adults [49]. However, IL-15 receptor signalling subunit IL2RB expression was 80% lower in older muscle, suggesting that the intracellular pro-myogenic effects of IL-15 may be blunted in the elderly. In a separate study, plasma IL-15 concentrations were found to be lower in sarcopenic compared to non-sarcopenic older adults and inversely correlated with sarcopenic state and body mass index (BMI) [50]. With regard to adiposity and IL-15 expression, obese individuals present with lower serum IL-15 than normal-weight individuals and IL-15 knockout in mice results in weight gain [139]. Interestingly, the aforementioned study that examined the effect of recombinant IL-15 administration in rats reported a decline in WAT mass [138].

It is important to note that levels of tissue IL-15 mRNA are not necessarily concordant with IL-15 protein expression and secretion, on account of inefficient translation due to multiple AUG initiation sites in the 5′-untranslated region [140]. Furthermore, alternative splicing produces two mature isoforms, only one of which follows the secretory pathway [141]. The seemingly contradictory observations of increased IL-15 mRNA expression in ageing and reduced circulating IL-15 abundance in sarcopenia may therefore demonstrate a transcriptional attempt to offset some impaired translational or secretory capacity that may occur with inflammaging, in an attempt to mitigate the catabolic effects of declining circulating IL-15.

Collectively, the literature suggest that IL-15 expression and function is tightly regulated at multiple levels and plays a significant role in the preservation of skeletal muscle mass. Its significance appears greatest within an atrophic, inflammatory state, such as that commonly observed with ageing, where it may have therapeutic potential through amelioration of processes involved in protein degradation.

### Adiponectin

Adiponectin is the most abundant peptide secreted by adipocytes and plays a key role in energy homeostasis [142–144]. Adiponectin functions through the binding of the adaptor protein APPL1 (phosphotyrosine interacting with PH domain and leucine zipper 1), to the AdipoR1 and AdipoR2 receptors, and promotes interaction between the insulin receptor (IR) and its substrates IRS1/2, consequently priming the phosphatidylinositol-3-kinase (PI3K) pathway [145–147]. In skeletal muscle, adiponectin enhances p38 mitogen-activated kinase (MAPK)/peroxisome proliferator-activated receptor gamma coactivator 1-alpha (PGC-1α) signalling and mitochondrial biogenesis [148, 149], and stimulates activation of adenosine monophosphate-activated protein kinase (AMPK) and acetyl-CoA carboxylase [150–152].

Unlike many adipocyte-secreted factors, adiponectin expression and secretion are greatest from healthy adipocytes of lean individuals and are inhibited by pro-inflammatory agents [124, 153, 154]. Accordingly, adiponectin levels decrease with obesity and type 2 diabetes mellitus (T2D) and correlate positively with insulin sensitivity and negatively with visceral adiposity [155–157]. Pertinently, circulating adiponectin is lower in sarcopenic than non-sarcopenic older adults [51] but does not differ between physical activity-matched non-sarcopenic older and younger adults, suggesting that it is not ageing per se driving reduced adiponectin concentration, but rather an age-associated decline in habitual physical activity level [52]. Conversely, circulating adiponectin was found to be higher in cardiovascular disease (CVD) patients with sarcopenia than without. However, the sarcopenic CVD group were predominantly female (66% female compared to a 28% female non-sarcopenic group) who typically present with higher adiponectin concentrations [158–160]. Nevertheless, some studies have suggested that elevated adiponectin may predict reduced muscle strength and function in older adults [161, 162]. Therefore, the higher levels of adiponectin in sarcopenic individuals reported in some studies could reflect a compensatory adaptation. Such a compensatory induction could be an attempt to preserve the p38 MAPK translational initiation pathway, the activation of which is dysregulated in human ageing [163], thus protecting against age-related impairment of protein synthetic pathways.

Globular adiponectin activates muscle satellite cells, promotes their motility, induces muscle gene expression including myosin heavy chain (MHC), myogenin, and p21, and drives myoblasts to exit the cell cycle, promoting differentiation [53–55]. Adiponectin overexpression increased regenerating myotubes in a mouse model of ageing and heart failure [164]. Adiponectin was found to accumulate on the myofibre plasma membrane and intracellularly co-localised with endosomes positive for the multivesicular/exosome marker cluster of differentiation-63 in regenerating, but not intact, fibres. However, in T-cadherin-null mice myotube regeneration was not increased by adiponectin overexpression, suggesting an essential role of T-cadherin in mediating these effects [164]. The authors demonstrated that both globular adiponectin and a small molecule mimetic (GTDF) induced differentiation and supressed MURF1 and MAFbx in various atrophy models, preventing the loss of myotube area [165]. Crossing degenerative muscle-diseased mice with mice overexpressing adiponectin improved expression of the myogenic differentiation markers myogenic regulatory factor-4 and myogenin, while reducing muscle inflammation and oxidative stress, resulting in higher muscle force and endurance [166]. Although this appears to contradict findings from human studies [161, 162], it supports the aforementioned possibility of a protective role of adiponectin on skeletal muscle mass in dysregulated states.

Confoundedly, stimulation of C2C12 cells with an adiponectin receptor agonist (AdipoRon) reduced cellular protein content, myotube diameter, and myotube multinucleation in a dose-dependent fashion (5–20 μM). However, the relevance of the dose utilised to physiologically elevate adiponectin concentrations is not well understood [167]. In mice, the extensor digitorum longus (fast twitch), but not soleus (slow twitch) muscle, exhibited increased adiponectin expression with ageing, though adiponectin receptor expression was unchanged [167]. The authors proposed that excessively high circulating adiponectin may induce skeletal muscle atrophy within fast-type muscle. However, neither serum AdipoRon nor adiponectin were measured in this study, limiting extrapolation and comparison to humans. Given that adiponectin activates AMPK, and AMPK activity has an inhibitive effect on mammalian target of rapamycin (mTOR), which plays a central role in protein synthesis, it is plausible that highly elevated adiponectin could exert negative effects on muscle mass regulation by excessive activation of AMPK [168–171]. Thus, it has been speculated that there is a healthy circulating adiponectin range required to maintain normal adiponectin signalling, though this purported range has not yet been clearly defined [171].

### Leptin

Leptin is a cytokine-like hormone that is abundantly secreted by adipocytes but is also expressed in skeletal muscle [172]. Leptin conveys satiety-promoting effects and plays a significant role in the regulation of energy balance and body mass, as well as lipolysis and insulin sensitivity, which has been reviewed elsewhere [173–176]. Circulating leptin concentration increases with obesity, correlates with BMI and adipose tissue mass [81, 177–180], and is reduced with weight loss [181–184]. Pertinent to the regulation of skeletal muscle mass, leptin administration increases hindlimb muscle mass and fibre size in aged mice [56], and prevents muscle atrophy in leptin-deficient mice [57]. Mechanistically, the binding of leptin to the long form of its receptor, activates Janus kinase 2 (JAK2), subsequently promoting PI3K activity and phosphorylation of protein kinase B (Akt) and p38 MAPK [58, 185–188]. Leptin promotes C2C12 myoblast proliferation but suppresses expression of myogenin and myoblast determination protein (MyoD), which is mediated by JAK-STAT (signal transducer and activator of transcription) and MAPK pathways [59]. Despite the propensity for leptin to promote pathways involved in muscle regulation, its obesity-mediated upregulation is associated with resistance to its action [176, 189–191]. Central leptin receptor expression may be reduced as a direct result of increased leptin abundance [192–194]. Concordantly, skeletal muscle protein expression of the long leptin receptor is lower in adults with obesity, suggesting one mechanism by which peripheral leptin resistance may occur [60].

While circulating leptin is elevated in animal models of ageing in the absence of obesity [195], it has been reported to decline in elderly adults with severe frailty, but not in community-dwellers [61]. This decline may reflect not only the low abundance of adipose tissue often seen in frail older adults but also the diminishing mass of skeletal muscle, which is also a significant source of leptin [196–198]. Conversely, in community dwelling older adults, appendicular skeletal muscle mass and thigh muscle cross-sectional area have been negatively associated with plasma leptin concentration, even after adjustments for bodyweight or body fat percentage [62, 63]. In these studies, circulating leptin abundance was greater in those presenting with either sarcopenia or obesity, and greater still in individuals presenting with sarcopenic obesity. Interestingly, physical inactivity in the form of bed rest, which is often characteristic of frail older adults, also increases leptin levels independent of changes in fat mass [199]. It could be argued that physical inactivity increases leptin secretion in an attempt to preserve muscle mass, which may be ineffective in an aged-obese environment due to existing leptin resistance. Collectively, the literature suggests that both sarcopenia and obesity may promote a hyperleptinaemic environment that drives peripheral leptin resistance via the downregulation of its receptor. Thus, obesity may amplify resistance to the muscle-preserving effects of leptin in ageing and contribute to the sarcopenic-obese state.

### Lipocalin 2

Lipocalin 2 (Lcn2) is a secretory glycoprotein first identified in human neutrophils and later noted for its abundant expression in adipocytes [200–202]. Both Lcn2 and its cell surface receptor (24p3R) have also been found to be expressed in skeletal muscle [64, 203]. Lipocalins bind and transport small hydrophobic molecules such as fatty acids, steroids, and retinol [200]. Lcn2 is induced by factors that drive insulin resistance and inflammatory stimuli, with transactivation by the pro-inflammatory transcription factor NF-κβ [65, 204]. Circulating Lcn2 is elevated with obesity and correlates with adiposity, adipose distribution, and inflammatory markers, though the significance of this relationship is contestable and not necessarily causal, with Liu et al. observing circulating Lcn2 to neither correlate with nor predict the incidence of insulin resistance or cardiovascular risk factors [66, 202, 205, 206].

Adipose Lcn2 mRNA and protein expression is greater in humans and rodents that are obese, compared to those of normal-weight [67, 200, 202]. Catalán et al. found plasma Lcn2 concentration was not affected by obesity; however, circulating Lcn2/matrix metalloproteinase (MMP) complex abundance and VAT MMP-2 and MMP-9 activity were elevated [67]. Given the role of the MMP system in adipocyte differentiation and SAT and VAT remodelling, this may implicate Lcn2 in the development of obesity [207–209]. On the other hand, it has recently been argued that rather than being causal of metabolic dysfunction, elevated Lcn2 may instead provide a protective mechanism to mitigate obesity-induced dysregulation and preserve pancreatic β-cell function [210]. Indeed, global Lcn2 knockout in mice was recently shown to accelerate age-dependent weight gain and visceral fat deposition in female mice, although curiously this was not the case in male mice [211].

Rebalka et al. investigated the role of Lcn2 in regenerating skeletal muscle and the effects of Lcn2 deletion [64]. While Lcn2 protein was lowly expressed in uninjured mouse skeletal muscle, its expression in Pax7^+^ muscle satellite cells was increased in response to cardiotoxin injury. In contrast, global Lcn2 knockout mice had reduced satellite cell activation and diminished muscle regeneration, with decreased embryonic MHC expression and smaller myofibre areas. However, the effects on adipose tissue were not studied. Finally, consistent with the role of Lcn2 in MMP regulation, Lcn2 knockout mice displayed greater fibrosis and lower MMP-9 activity during muscle regeneration.

The relationship between adipose-derived Lcn2 and skeletal muscle regulation is not yet fully understood, with reports of global Lnc2 knockout in C57BL/6 mice conferring both protection from, and potentiation of, diet- and ageing-induced metabolic dysregulation [212–214]. Initial investigations have shown that anti-diabetic drugs can counteract obesity-upregulated Lcn2 expression and circulating abundance in rats, T2D mice, and humans with T2D [200, 206]. However, reports of acute, but not chronic, exercise elevating circulating Lcn2 abundance in obese adults suggests that the relationship between Lcn2 and metabolic health is complex [215, 216]. Furthermore, it is not yet clear how modulation of circulating Lcn2 might impact skeletal muscle regulation. Therefore, studies employing muscle and adipose tissue-specific deletion as well as overexpression or administration of recombinant Lcn2 within rodent models of ageing and muscle wasting are warranted.

### Myostatin

Myostatin is a transforming growth factor-beta (TGF-β) superfamily member that negatively regulates muscle mass and whose disruption produces hyper-muscularity [68]. Myostatin signals through activin-responsive type II receptors (ACTRIIA and ACTRIIB) and the type I receptors, activin-like kinases, triggering activation of small mothers against decapentaplegic (SMAD)2 and SMAD3 transcription factors, resulting in the inhibition of hypertrophic and activation of atrophic pathways [69, 217–221]. Recombinant myostatin impairs myogenesis through suppression of satellite cell activation and myoblast proliferation and differentiation, while genetic inactivation impairs myotube formation in vitro [70–72, 222, 223]. Myostatin expression and abundance is reportedly, though not universally, elevated with obesity, advancing age and muscle wasting, and decreased by weight loss [73, 224–229].

Whether the deleterious association of obesity and myostatin expression reflects an effect of excessive adipose tissue per se, or the product of an obesogenic muscle environment, remains unclear. Similarly, exercise is a potent suppressor of myostatin expression [230–232], and thus physical activity status adds a further confounding variable that is not always controlled for. Nevertheless, primary myotubes generated from morbidly obese individuals exhibit ~ 2-fold greater myostatin protein content and ~ 3-fold greater secretion than myotubes from lean donors, and mRNA expression is similarly elevated in morbidly obese muscle [73, 233].

Myostatin is also expressed in adipose tissue; however, data from ob/ob mice suggest mRNA expression is 50–100-fold lower than in skeletal muscle [234]. Importantly, myostatin expression was assessed in the tibialis anterior, a muscle comprised almost exclusively of fast fibres [235]. Since MHC IIb (a fast twitch myosin) expression correlates with myostatin mRNA expression, this may in part explain the magnitude of difference reported between muscle and adipose myostatin expression [236]. Nevertheless, both myostatin and ActRIIB mRNA expression were 50–100-fold higher in ob/ob than wild-type adipose tissue. Conversely, despite elevated serum abundance, myostatin expression in SAT and VAT was not different between age-matched severely obese, lean, and overweight humans [237]. It is plausible that the contradictory findings between obese human and murine adipose tissue myostatin expression reflect fundamental differences between human and murine adipose expression profiles and brown adipocyte abundance [238, 239].

Adipose tissue-specific deletion of myostatin in mice fed a HFD did not affect muscle weight nor body composition; however, whole-body knockout partially supressed HFD-induced fat accumulation [77, 240]. Conversely, muscle-specific inhibition of myostatin signalling increased lean mass and decreased fat mass in both chow and high-fat diets [77]. These studies suggest that secretion and signalling of myostatin in skeletal muscle influence the regulation of muscle and adipose mass. However, myostatin secretion and its signalling in adipose tissue have little impact on either adipose or muscle regulation, reflecting its vastly lower expression in adipose tissue.

Ageing appears to upregulate myostatin expression independent of adiposity, which if translated to increased myostatin protein secretion could contribute to sarcopenia by promoting atrophic and inhibiting hypertrophic pathways via canonical TGF-β signalling. Muscle myostatin mRNA and protein expression was 2.0-fold and 1.4-fold higher, respectively, in older than younger males [224] and myostatin mRNA expression was 56% greater in elderly than young women [74]. Older males matched with younger males for total- and lean-body mass and body fat percentage tended to have higher basal muscle myostatin mRNA expression and significantly greater muscle myostatin protein content [75]. Whether elevated myostatin expression with advancing age is a function of ageing per se or a product of age-associated physical inactivity, systemic inflammation, or nutritional status remains poorly investigated. Interestingly, muscle myostatin mRNA was found to be lower in both ambulatory and non-ambulatory elderly women than young women [76]. Myostatin protein content was greatest in the old ambulatory group, with no difference in plasma concentrations between groups. Similarly, higher concentrations of serum myostatin have been reported in non-frail compared to frail nursing home residents, and its abundance was increased in male residents, of both frail and non-frail status, following chronic exercise [241]. Evidently, further research is necessary to characterise and delineate between the effects of advancing age, adiposity, and physical inactivity on human myostatin expression and function in vivo.

## Long non-coding RNAs: emerging roles in skeletal muscle regulation

Only ~ 1% of the human genome is translated into proteins; however, much is transcribed into non-protein-coding RNAs [242, 243]. Of these, lncRNAs, with transcripts of > 200 nucleotides, are the largest group and have been implicated in the regulation of RNA transcription, splicing, and trafficking, miRNA regulation, and RNA stability [84, 244–248]. lncRNAs have been implicated in obesity and in obesity-associated disorders including muscle wasting [249, 250]. Sequencing of SAT from females with and without obesity identified 86 lncRNAs that were differentially expressed [251]. Skeletal muscle from individuals with inclusion body and anti-Jo-1-associated myositis, a collection of diseases characterised by chronic muscle inflammation and weakening, revealed a similar number of differentially expressed lncRNAs, relative to healthy controls [78]. Of these, 16 lncRNAs were differentially expressed in both myositis groups, including H19, metastasis-associated lung adenocarcinoma transcript 1 (MALAT1), plasmacytoma variant translocation 1 (PVT1), and long non-coding myoblast determination protein (lncMyoD), which have been previously characterised and implicated in skeletal muscle regulation [84, 252, 253] (Table 1).

### H19

H19 is upregulated during myoblast differentiation and its inhibition impairs skeletal muscle differentiation [254]. Pertinently, two miRNAs encoded by H19 exon1, miR-675-3p and miR-675-5p, which are induced during differentiation, can rescue the effects of H19 depletion through suppression of the TGF-β/bone morphogenetic protein pathway. H19 expression in SAT and VAT negatively correlates with BMI; however, whether an obesity-associated decline in adipose H19 affects the regulation of skeletal muscle mass remains to be investigated [255]. Given the aforementioned differential skeletal muscle expression of H19 in myositis patients, its potential involvement in age-related muscle wasting also warrants investigation.

### MALAT1

MALAT1 expression increases during differentiation of primary human myoblasts and its knockdown is associated with impaired proliferation and differentiation [79, 256]. Treatment of mice with recombinant myostatin drastically reduced MALAT1 expression, which given the positive impact of myostatin deficiency on muscle function with ageing suggests MALAT1 may be an important downstream target of myostatin and regulator of myogenesis with ageing that deserves examination [256–258]. MALAT1 can competetively bind miR-133, de-repressing the transcription factor serum response factor (SRF) in myoblasts, facilitating upregulation of muscle-specific gene expression [79]. Conversely, it has been reported that MALAT1 is downregulated during early myogenesis and regulates differentiation through modulation of MyoD [80]. The impact of adiposity on MALAT1 expression in skeletal muscle and on myogenesis has not yet been explored.

### PVT1

PVT1 is activated during muscle atrophy, affecting myofibre size, apoptosis, and mitochondrial function [252]. Downexpression of PVT1 in muscle induces resistance to atrophic processes in response to a denervation model of muscle wasting, by modulating apoptosis and autophagy. PVT1 is implicated in a variety of disease states characterised by inflammation, including obesity where its expression in murine adipose tissue is increased and promotes adipogenesis [82] and in osteoarthritis, where its suppression may ameliorate disease progression through the repression of catabolism and inflammation [83, 259]. However, PVT1 in ageing has not yet received much attention and its role in the cross talk between adipose tissue and skeletal muscle remains unknown.

### lncMyoD

Encoded next to the MyoD gene, lncMyoD is activated by MyoD during myoblast differentiation and binds to insulin-like growth factor (IGF) mRNA-binding protein 2 (IMP2), blocking IMP-mediated shuttling of proliferation-promoting RNAs, inhibiting their translation and thus proliferation [84, 85]. The MyoD-lncMyoD-IMP2 pathway demonstrates a mechanism involved in the proliferation-inhibitive effects of MyoD, promoting a permissive state for differentiation. In mice, skeletal muscle lncMyoD expression is upregulated during models of disuse atrophy including denervation, casting, and tail suspension, but not in models of systemic muscle wasting including dexamethasone administration, cancer cachexia, and fasting [260]. Given the prevalence of physical inactivity and immobilisation amongst the elderly, such findings warrant investigations into the role and therapeutic potential of lncMyoD in human muscle ageing.

## MicroRNAs: novel candidate factors in adipose-muscle cross talk

The non-coding miRNAs are well established as key mediators of gene expression through post-transcriptional downregulation, translational repression, and deadenylation-dependent decay and are abundant within EVs [261–268]. Most miRNAs are first transcribed into primary miRNAs (pri-miRNAs), processed by the RNase III enzymes Drosha, forming precursor miRNAs (pre-miRNAs), and Dicer, yielding mature miRNAs [269–271]. Recent reviews and systematic analyses of microarrays and next-generation sequencing have highlighted a plethora of miRNAs that are differentially expressed in skeletal muscle and/or plasma with ageing and muscle wasting, though the functional roles of many of these novel miRNAs remain to be elucidated [89, 272–275]. Similarly, within metabolic tissues and the circulation, numerous miRNAs have been implicated in the pathological development of obesity and may serve important functions in metabolic organ cross talk [276–281].

Whether obesity exacerbates sarcopenia through miRNA-mediated adipose-muscle cross talk is unclear. However, the capacity for adipose tissue to mediate metabolic cross talk with skeletal muscle via miRNAs is evident. Indeed, Wang et al. showed that miR-130b is secreted from adipocytes during adipogenesis; its WAT mRNA expression is increased with obesity; its circulating abundance is elevated in human and murine obesity, correlates with BMI, and predicts the metabolic syndrome; and it is able to target muscle cells, supressing expression of its target gene, PGC-1α [282].

While direct investigations of possible miRNA-mediated cross talk in the context of age-related muscle wasting and obesity remain to be undertaken, separate investigations have uncovered differentially expressed miRNAs common to both ageing and obesity, including miR-31, miR-223, and miR-33a [283, 284]. Such miRNAs may present candidate mediators of the adverse effects of obesity on muscle mass regulation (Table 1).

### miR-31, miR-223, and miR-33a

Administration of leptin to aged mice increased muscle mass and expression of miR-31 and miR-223, which are known to be elevated during muscle regeneration and repair [56]. Given the aforementioned (“Leptin” section) potential role of leptin in the regulation of skeletal muscle mass, this suggests a channel by which miRNAs may be involved in mediating a beneficial effect of leptin in ageing muscle [56, 284].

miR-33a is expressed in both human skeletal muscle and adipose tissue [285] and encoded within sterol regulatory element-binding protein 2, a transcription factor involved in lipid biosynthesis and trafficking [286]. The circulating level of miR-33a is lower in older than younger adults in both the presence and absence of insulin resistance [283]. It has been shown that miR-33a knockout mice develop obesity with increased proliferation of pre-adipocytes, increased lipid uptake, and impaired lipolysis [287, 288]; however, the effect of obesity per se on miR-33a expression and abundance is not known. Administration of a miR-33a mimetic to primary duck myoblasts impaired proliferation, while a miR-33a inhibitor enhanced proliferation, and it was demonstrated that miR-33a may directly target IGF-1, follistatin, and cyclin-D1 to inhibit myoblast proliferation by suppressing the PI3K/Akt/mTOR signalling pathway [289]. Taken together, these data suggest that miR-33a is essential for normal adipocyte development and function; however, its excessive abundance may impair myogenesis. Thus, the declining levels of circulating miR-33a seen with ageing may act to offset the otherwise declining myogenic capacity of skeletal muscle. However, more research is needed to unravel the role of miR-33a in regulating muscle mass in the context of ageing and obesity.

### MyomiRs

Numerous muscle-enriched miRNAs (myomiRs) have been implicated in myogenesis through their involvement with myogenic regulatory factors [290–293]. Using microarrays, miR-133a and miR-133b, which promote myoblast proliferation through repression of SRF [86], were found to be downregulated in muscle from healthy older men [89]. Conversely, elevated levels of pri-miRNA-1-1, pri-miRNA-1-2, pri-miRNA-133a-1, and pri-miRNA-133a-2 were found in the muscle from elderly men, relative to younger men [90]. However, these differences did not exist at the mature miRNA level, suggesting differential processing of pri- and pre-miRNAs with ageing, although dissimilar to prior findings, Drosha and Dicer protein or mRNA expression were not different between young and older men [90, 294].

Combined injection of miR-1, miR-133, and miR-206 accelerates muscle regeneration in vivo, induces myogenic expression, and promotes myoblast differentiation in vitro [87, 293]. miR-1 promotes myogenesis through targeting of the muscle gene transcriptional repressor histone deacetylase 4 (HDAC4), and its expression and circulating abundance are suppressed by HFD in mice [295, 296]; however, skeletal muscle miR-1 mRNA expression was elevated in a murine model of progeroid ageing [88]. miR-206 promotes myoblast differentiation and directly represses follistatin-like-1 and utrophin; however, its mRNA expression was also found to be elevated in aged mouse muscle [56, 91]. Pertinently, miR-206 expression in plasma was found to be downregulated 0.5-fold in children with overweight/obesity relative to normal-weight children, suggesting a potential mechanism by which obesity might adversely affect myogenesis independent of ageing [297]. Similarly, obesity is associated with downregulation of miR-133a-3p in human SAT [298] and in mice HFD-induced obesity downregulates miR-133b in WAT [299]. Conversely, HFD-induced obesity upregulates miR-133a in mouse muscle [295]; however, the functional significance of these observations in the context of regulating skeletal muscle mass remains unclear.

It is growing ever more apparent that miRNAs play vital roles in myogenesis and preliminary studies have identified both interacting and diverging effects of obesity and ageing on their expression and function. The lack of consensus between these studies highlights the need for further research, with an emphasis on translational studies to confirm findings from sequencing and microarray studies, to delineate the impact of ageing and obesity per se on the miRNA-mediated regulation of skeletal muscle mass. The subsequent identification and confirmation of differentially regulated adipose-muscle cross talking miRNAs will doubtless present novel potential therapeutic targets for muscle wasting conditions.

## Conclusions and future directions

The regulation of skeletal muscle mass with ageing is complex and delicately balanced, involving many secreted molecules (including proteins and RNAs), which often display pleiotropic effects. Many of these factors appear perturbed by excessive adiposity, but effects are confounded by age, physical activity status, and comorbidities, frequently resulting in conflicting findings and limited reproducibility. Indeed, the potent beneficial effects of exercise on muscle-remodelling are a stark contrast to the rapid deterioration of muscle mass and function observed during periods of physical inactivity and immobilisation, which feature in the lives of some, but not all, elders. It is imperative therefore that every effort is taken to control for physical activity status when conducting human studies. Advances in proteomics and transcriptomics are enabling greater investigation of the secretomes of human muscle and adipose tissue in healthy, dysfunctional, and senescent states. There is a need, however, to extend the application of these techniques beyond the cross-sectional studies that have identified the differences in these secretomes, and to apply them to longitudinal studies of human ageing. Similarly, co-culture techniques using human primary cells from well-characterised donors are improving our understanding of these factors across the spectrums of health and ageing and offer greater relevance to the human condition. Finally, advances in techniques to investigate extracellular vesicles, to identify novel non-coding RNAs, and to establish their functional significance in regulating skeletal muscle mass may identify novel therapeutic targets for preserving muscle mass with ageing and increasing adiposity and may enhance our understanding of the sarcopenic-obese phenomenon.

## Abbreviations

ACTRIIB: Activin receptor type IIB
Akt: Protein kinase B
AMPK: Adenosine monophosphate kinase
APPL1: Adaptor protein, phosphotyrosine interacting with PH domain and leucine zipper 1
BMI: Body mass index
CVD: Cardiovascular disease
HDAC4: Histone deacetylase 4
HFD: High-fat diet
IFN-γ: Interferon gamma
IGF: Insulin-like growth factor
IL: Interleukin
IMP2: Insulin-like growth factor 2 mRNA-binding protein 2
IRS: Insulin receptor substrate
JAK: Janus kinase
Lcn2: Lipocalin-2
lncMyoD: Long non-coding myoblast determination protein
lncRNA: Long non-coding RNA
MAFbx: Muscle atrophy F-box gene/atrogin-1
MALAT1: Metastasis-associated lung adenocarcinoma transcript 1
MHC: Myosin heavy chain
miRNA: MicroRNA
MMP: Matrix metalloproteinase
mTOR: Mammalian target of rapamycin
MuRF-1: Muscle ring finger-1
MyoD: Myoblast determination protein
NF-κβ: Nuclear factor-kappa beta
P38 MAPK: P38 mitogen-activated kinase
Pax7: Paired box 7
PGC1-α: Peroxisome proliferator-activated receptor gamma coactivator 1-alpha
PI3K: Phosphatidylinositol-3-kinase
Pre-miRNA: Precursor-microRNA
Pri-miRNA: Primary-microRNA
PVT1: Plasmacytoma variant translocation 1
SASP: Senescence-associated secretory phenotype
SAT: Subcutaneous adipose tissue
SMAD: Small mothers against decapentaplegic
SRF: Serum response factor
STAT: Signal transducer and activator of transcription
T2D: Type 2 diabetes mellitus
TGF-β: Transforming growth factor-beta
TNF-α: Tumour necrosis factor-alpha
VAT: Visceral adipose tissue
WAT: White adipose tissue
